# Reconstruction of *Litopenaeus vannamei* Genome-Scale Metabolic Network Model and Nutritional Requirements Analysis of Different Shrimp Commercial Varieties

**DOI:** 10.3389/fgene.2021.658109

**Published:** 2021-05-12

**Authors:** Chenchen Gao, Jiarui Yang, Tong Hao, Jingjing Li, Jinsheng Sun

**Affiliations:** ^1^Tianjin Key Laboratory of Animal and Plant Resistance, College of Life Sciences, Tianjin Normal University, Tianjin, China; ^2^Tianjin Fisheries Research Institute, Tianjin, China

**Keywords:** genome-scale metabolic network, feed, biomass, nutrient requirement, *Litopenaeus vannamei*

## Abstract

As an important tool for systematic analysis, genome-scale metabolic network (GSMN) model has been widely used in various organisms. However, there are few reports on the GSMNs of aquatic crustaceans. *Litopenaeus vannamei* is the largest and most productive shrimp species. Feed improvement is one of the important methods to improve the yield of *L. vannamei* and control water pollution caused by the inadequate absorption of feed. In this work, the first *L. vannamei* GSMN named *i*GH3005 was reconstructed and applied to the optimization of feed. *i*GH3005 was reconstructed based on the genomic data. The model includes 2,292 reactions and 3,005 genes. *i*GH3005 was used to analyze the nutritional requirements of five different *L. vannamei* commercial varieties and the genes influencing the metabolism of the nutrients. Based on the simulation, we found that tyrosine-protein kinase src64b like may catalyze different reactions in different commercial varieties. The preference of carbohydrate utilization is different in various commercial varieties, which may due to the different expressions of some genes. In addition, this investigation suggests that a rational and targeted modification in the macronutrient content of shrimp feed would lead to an increase in growth and feed conversion rate. The feed for different commercial varieties should be adjusted accordingly, and possible adjustment schemes were provided. The results of this work provided important information for physiological research and optimization of the components in feed of *L. vannamei*.

## Introduction

*Litopenaeus vannamei*, also known as South American white-leg shrimp, is naturally distributed in the pacific coastal waters from northern Peru to Mexico. It has become the world’s largest and most productive shrimp species (Fan and Li, 2019). With the increasing demand for shrimps and its expansion, the *L. vannamei* breeding industry faces many challenges. In intensive culture mode, a large amount of nitrogen, phosphorus, and other nutrients in the low-quality feed with unreasonable nutrition formulation are directly discharged into the water body without being absorbed by *L. vannamei*, which is an important inducement of water pollution and shrimp disease (Briggs and Fvnge-Smith, 1994). Therefore, it is an essential issue to reasonably allocate the proportion of nutrients in feed in order to improve the growth and feed conversion rate of shrimp, so as to control the ammonia nitrogen output.

To solve this problem, an in-depth and systematic study on the appropriate proportion of nutrients for *L. vannamei* is needed to construct the feed with balanced nutrition. Since the 1980s, scientists have successively researched on the nutrition, feed formulation, feed quality detection, and feed preparation technology of primary aquatic organisms (Kangsen, 2020). Shrimps need various protein, lipid, carbohydrate, vitamins, and minerals as nutrients to maintain their physiological and growth requirements (Qihui and Qicun, 2005). At present, the nutrition research of *L. vannamei* mainly focuses on the proportion of nutrients needed for its growth through trial-and-error experiments. Various substances have been tried as feed additives to test their effects on improving the growth performance of *L. vannamei*, including butonlin onion extract (Munaeni et al., 2019), succinic acid (Duan et al., 2018), β-glucan (Li et al., 2019), yeast hydrolysate (Jin et al., 2018), and large seaweeds (Niu et al., 2018). These studies focus on some specific compounds but lack systematic analysis, which limit the comprehensive exploration and analysis of the nutritional requirements of *L. vannamei*.

Since the first genome-scale metabolic network (GSMN) model was proposed in 1999 (Edwards and Palsson, 1999), GSMN has become the main tool to systematically study the metabolic system. GSMN is reconstructed based on genomic sequencing, relevant databases, and experimental data to represent the relationship among genes, proteins, and reactions. It mathematically describes the metabolic reactions based on stoichiometry and mass balance (Kim et al., 2008; Thiele and Palsson, 2010). Moreover, it can be used to simulate the metabolic flux of the whole metabolic system through flux balance analysis (FBA) and other algorithms (Orth et al., 2010).

To date, nearly 200 manually modified high-quality GSMNs have been reconstructed, which cover bacteria, virus, archaea, and many eukaryotes (Gu et al., 2019). The classic simulation of GSMN is to calculate the maximum growth rate of the target species, which can be used to analyze the biological nutrient intake and provide guidance for metabolic engineering experiments. The analysis of biological nutrient intake has been applied to multiple species. For example, Feist et al. (2007) predicted the absorption and utilization of 312 nutrients in *Escherichia coli* culture medium with *E. coli i*AF1260, including 174 carbon sources, 78 nitrogen sources, 49 phosphorus sources, and 11 sulfur sources. Similarly, Oh et al. (2007) predicted the absorption and utilization of 379 nutrients in *Bacillus subtilis* culture medium with a *B. subtilis* GSMN. However, the GSMN for *L. vannamei* has still not been reconstructed due to the lack of genomic data. Fortunately, the research team of Jianhai Xiang and Fuhua Li completed the whole-genome sequencing of *L. vannamei* in 2019 (Zhang et al., 2019). Subsequently, various databases collected relevant information about the *L. vannamei* genome, which provided a database for the reconstruction of GSMN and systematic analysis of the metabolic process in *L. vannamei*.

In this work, we reconstructed the first *L. vannamei* GSMN based on its genomic data. The nutritional compositions of five *L. vannamei* commercial varieties, including Lutai, Riyekuai, Kehai, Guangtai, and Puruiying, were detected. Based on the nutritional components, the biomass reactions were constructed for these commercial varieties and added into the model to make it capable of simulating the growth of cells. The reconstructed GSMN was used to analyze the nutritional requirements of different commercial varieties. Based on this investigation, the important genes affecting the utilization of nutrients were discussed and the suggestions for feed improvement were proposed, which laid the foundation for the growth research and optimization of feed formulation for *L. vannamei*.

## Materials and Methods

### Reconstruction of the Genome-Scale Metabolic Network

The reconstruction of the GSMN almost follows the standard protocol proposed by Thiele and Palsson (2010). The reconstruction process is composed of draft reconstruction, refinement of network, adding biomass reaction, conversion of network to model, and model evaluation.

#### Draft Reconstruction

The data required for the draft reconstruction of the GSMN were downloaded from Kyoto Encyclopedia of Genes and Genomes (KEGG) database, including the information of genes, metabolic reactions (ID, name, main reaction equation, whole reaction equation, and direction), enzymes, metabolites (ID, name), pathways, and subsystems. The GSMN was draft reconstructed after all the data were sorted out and mapped.

#### Refinement of Network

##### Supplement of the missing information

The missing information in the network mainly includes balance of reaction, main reaction, pathway, and subsystem for some reactions. For the unbalanced reactions, their equations were balanced according to element conservation and charge conservation principles. The lacking main reaction was added according to the pathway map and “rclass” in KEGG database. The pathway of the reaction is determined by the pathway of metabolites involved in the reaction. After pathway information has been added, the subsystem information can be paired to the corresponding reaction through the pathway–subsystem relationship downloaded from KEGG database to make the reaction information more comprehensive.

##### Chiral standardization of metabolites

In the KEGG database, different molecular conformations of the same metabolite were assigned different IDs. Taking glucose as an example, there are three IDs for glucose in KEGG, namely, C00031 (D-glucose), C00267 (alpha-D-glucose), and C00221 (beta-D-glucose). D-glucose is a molecule with uncertain chiral properties. In order to prevent the identification confusion caused by different IDs of the same compound in the metabolic network, the D-glucose in the reaction equation was changed to alpha-D-glucose because glucose in animal cells tends to exist in the form of alpha chirality.

##### Removing of redundant reactions

Some redundant reactions were deleted to simplify the network structure and ensure the reasonable distribution of metabolic flux. Firstly, when the total reaction and the step reactions exist at the same time and the step reaction has no branch, the total reaction was retained and the step reactions were deleted. Secondly, the general reactions were deleted, in which the reactants are a class of substances rather than specific metabolites. Thirdly, the uncompleted reactions and the reactions with coefficient “m” or “n” were deleted. Finally, the redundant reactions occurred because the chiral standardization of metabolites was removed from the network.

##### Gap filling

In the draft reconstruction, terminal metabolites were ubiquitous due to the lack of gene annotation and incomplete understanding of the metabolic systems in an organism. The existence of terminal metabolites may lead to breakpoints in some metabolic pathways, which result in the simulation error of some metabolic functions. To reduce the terminal metabolites and breakpoints, we calculated some reactions from KEGG database to fill the gaps in the network. The gaps were filled in two scales: pathway scale (Hao et al., 2010) and global scale (Hao et al., 2012). Taking the global scale gap filling as an example, the global network is first divided into several weakly connected components (WCCs). In all reactions in the KEGG database, the reaction that can connect two WCCs into one is screened out, which is called a “gap reaction.” Adding gap reactions into the network can fill the network gaps.

##### Adding transport and exchange reactions

The addition of transport and exchange reactions is based on the nutritional requirements of *L. vannamei*. Exchange reactions are responsible for taking up extracellular metabolites (nutrients) from the environment or transporting extracellular metabolites (secretory products) into the environment, while transport reactions are in charge of transporting metabolites between intracellular and extracellular parts. Considering the nutritional requirement of *L. vannamei*, we added the exchange and transport reactions for amino acids, carbohydrates, lipids, vitamins, mineral elements, and cofactors.

##### Cellular compartments

The cellular location in the model was divided into two compartments, cytoplasmic [cytosol, represented by (c)] and extracellular [extracellular, defined by (e)]. Cellular compartment information was added for all the reactions and metabolites in the network. The same metabolite in different locations is considered as different metabolites.

##### Verification of gene–enzyme–reaction relationship

The relationships between gene–enzyme and enzyme–reaction were all derived from the KEGG database. Using enzyme as a bridge, the relationships between gene–enzyme–reaction were connected. If multiple genes correspond to one reaction, intergenic relationships need to be determined. When the genes of a reaction correspond to different enzymes, their relationships are considered “or,” and if several genes encode the same enzyme, their relationships are considered “and” (Wang et al., 2020).

#### Addition of Biomass Reaction

##### Nutritional composition detection of *litopenaeus vannamei*

Five commercial varieties of healthy *L. vannamei* were taken from the Haitong lake culture farm. As we all know, muscle is the main edible part of *L. vannamei*. Meanwhile, metabolic processes mostly occur in the hepatopancreas. Therefore, muscle and hepatopancreas were sampled for detection. Here, 150–200-g samples from each variety were collected and sent to Beijing Institute of Nutritional Sources for nutritional composition detection using the standard method specified in “GB28050-2011 national food safety standard general principles for nutrition labeling of prepackaged food” (Liu and Ge, 2013). The contents of five saccharides, 20 amino acids, 10 trace elements, five nucleotides, and 25 fatty acids in the samples were tested.

##### Construction of biomass equation

The nutrient composition detection provides a category of substances to construct a biomass reaction, while the coefficient of these metabolites in the biomass reaction is calculated from their specific contents. The units of the contents from the nutritional detection are usually g/100 g, or mg/kg, which need to be converted to the model required unit “mmol/gDW.” gDW represents the cell dry weight, which is obtained by subtracting the mass of water from the total sample mass. To simulate the energy maintenance in the cell, cofactors including ATP, ADP, phosphate, and H^+^ were added. For *L. vannamei*, there is no energy maintenance data for reference, neither even in aquatic crustaceans or arthropods. Therefore, we referenced the coefficients in the biomass equation of mouse model *i*CHO1766 (Hefzi et al., 2016), which is reconstructed based on the combination of several existing models and physiological experiments. This is the closest model to *L. vannamei* among all the models including energy maintenance coefficients in biomass equation. Finally, the coefficients of energy maintenance cofactors were all set to be 29.8303.

#### Conversion of Network to Model

The network should be transformed to a mathematical model represented by a stoichiometric matrix to facilitate the mathematical calculation. The constraints of reactions should be limited for FBA analysis. The flux of reversible reactions was set to be (−1,000, 1,000) mmolgDW^–1^h^–1^ and that for irreversible reactions is (0, 1,000) mmolgDW^–1^h^–1^. The flux of transport reactions was set to be (−1,000, 1,000) mmolgDW^–1^h^–1^. For the nutrients that can be synthesized by the model, the flux of related exchange reactions was set to be (0, 1,000) mmolgDW^–1^h^–1^. The flux of the nutrients that need to be absorbed from the feeds was set to be (−5, 1,000) mmolgDW^–1^h^–1^, except for trace elements set to be (−1, 1,000) mmolgDW^–1^h^–1^. The transformation and simulation of the model were performed under MATLAB platform using COBRA toolbox (Heirendt et al., 2019).

#### Network Evaluation

The model was evaluated and revised by simulating the synthesis of 10 non-essential amino acids, 25 fatty acids, five carbohydrates, five nucleic acids, 10 mineral elements, and biomass to make it capable of synthesizing the substances that were mentioned above.

Taking glutamate as an example, the exchange reaction of glutamate was firstly set to be the objective function, and the maximum value of the objective function (that is, the maximum flux of the glutamate synthesis) was calculated with the model. If it was 0, it indicates that glutamate cannot be synthesized by the model, which means the model is flawed and needs to be revised. To find the defect of the model, the completeness of synthesis pathway for glutamate was checked. The lacking reaction(s) of the pathway was supplemented to the model and followed by a second simulation until the maximum value of the objective function is not 0, indicating that the model can synthesize glutamate.

In the biomass synthesis simulation, the biomass equation was set to be the objective function and the maximum value of the objective function (that is, the maximum synthesis flux of biomass) was calculated with the model. The detailed calculation process was supplied in Supplementary File 4.

### Nutrient Requirement Analysis

The biomass equation was set to be the objective function, and its synthesis rate was fixed to be 1 gDW^–1^h^–1^. The flux of exchange reactions for each nutrient was simulated by FBA, which is the optimal nutrient requirement of these nutrients for 1 g dry weight increase of *L. vannamei.* The nutritional requirements of the five commercial varieties of *L. vannamei* were calculated separately. For the purpose of giving constructive suggestions for fodder improvement, we compared the calculated results with literature and further analyzed the differences in the nutritional requirements among the five varieties. The detailed calculation process was supplied in Supplementary File 4.

## Results and Discussion

### Reconstruction of Genome-Scale Metabolic Network for *Litopenaeus vannamei*

#### Draft Reconstruction

The reaction, main reaction, enzyme, pathway files were downloaded from the KEGG database. Information including reaction ID, name, equation, enzyme number, gene, pathway ID and name, subsystem name was extracted and collated. The results are shown in Table 1.

**TABLE 1:** Information collected from KEGG database.

**Item**	**Count**
Reaction	11,464
Main reaction	7,191
Reaction with enzyme	10,127
Pathway	185
Subsystem	13

*KEGG, Kyoto Encyclopedia of Genes and Genomes.*

In 2019, the *L. vannamei* genome and related information were included in the KEGG database. To reconstruct the GSMN of *L. vannamei*, we downloaded the catalytic enzyme of *L. vannamei*, and 3,005 enzymes were obtained. Using enzyme number as a bridge, the genes of *L. vannamei* were matched to the reactions, and then the information of pathway and subsystem was integrated to each reaction, which composed the draft reconstruction of *L. vannamei* GSMN. The draft network contains 3,005 genes, 3,005 enzymes, 1,572 reactions, 108 pathways, and 12 subsystems.

#### Refinement of Reconstruction

The draft reconstruction was manually refined as described in the “Materials and Methods” section. Based on the conservation of elements and charges, 54 reactions were artificially balanced. Most of the reactions were balanced with the addition of H_2_O, H^+^, electron (e^–^), CH4, and ethylene. The main reactions, pathways, and subsystems of 441 reactions were supplemented with purine metabolism subsystem supplemented the most main reactions. After the standardization of chiral metabolites, 23 reaction equations were modified. Here, 100 redundant reactions were removed from the network, of which 23 were step reactions, 46 were uncompleted reactions, 13 were duplicated reactions due to the replacement of chiral metabolite, and 18 are reactions with coefficient “m” or “n.” After the manual revision, 1,472 reactions were included in the network.

The network was subsequently partitioned into WCCs, and gap filling was performed. There were 301 WCCs initially in the network. The gaps were filled first on the pathway scale and then on the global scale. In total, 207 and 415 reactions were added on the pathway and global scale, respectively. After gap filling, the number of WCCs in the network decreased from 301 to 162, indicating that the network gap filling enhanced the global connection of the network.

Based on the nutrient requirements of *L. vannamei*, transport and exchange reactions were added for 20 amino acids, 28 fatty acids, five saccharides, 15 vitamins, 10 mineral elements, and 12 cofactors. In total, 91 transport reactions and 91 exchange reactions were added. The subcellular localizations of metabolic reactions were all considered as cytoplasm, while the location of transport reactions spans both cytoplasm and extracellular part. The exchange reactions were all located at the extracellular part.

The refined network is composed of 3,005 genes, 2,261 reactions including 2,079 metabolic reactions, 91 transport reactions and 91 exchange reactions, 2,149 metabolites, including 2,056 cytosol metabolites and 93 extracellular metabolites, 128 pathways, 162 WCCs, and 12 subsystems.

#### Addition of Biomass Reaction

The samples of five commercial varieties sending to nutritional component detection are shown in Table 2. The average weight of shrimp was obtained by dividing the total weight by the number of shrimps sampled. According to the average weight, it can be seen that Riyekuai has the fastest growth rate. The growth rate of Guangtai was in the middle, while Lutai, Kehai, and Puruiying grow the slowest with comparable average weights. The results of nutritional component detection include the content of 20 amino acids, 25 fatty acids, 10 trace elements, five saccharides, and five nucleic acids (Supplementary File 1). The unit of each nutrient was converted to mmol/gDW, and then the content value is considered as its coefficient in the biomass equation. The coefficient maintenance substances such as ATP and nicotinamide adenine dinucleotide phosphate (NADPH) were referred to the mouse model. Finally, the biomass reaction was composed of the metabolites from the nutritional component detection and the maintenance substances. The biomass reactions for all the five commercial varieties of *L. vannamei* were constructed (Supplementary File 2).

**TABLE 2:** Samples for five commercial varieties of *L. vannamei*.

**Commercial variety**	**Quantity**	**Weight (g)**	**Average body length (cm)**	**Average weight (g)**
Lutai	21	188.84	9.12	8.99
Riyekuai	13	205.29	10.45	15.79
Kehai	25	169.24	7.96	6.77
Guangtai	16	199.75	10.18	12.48
Puruiying	29	181.68	7.54	6.26

#### Network Evaluation

**FIGURE 1:**
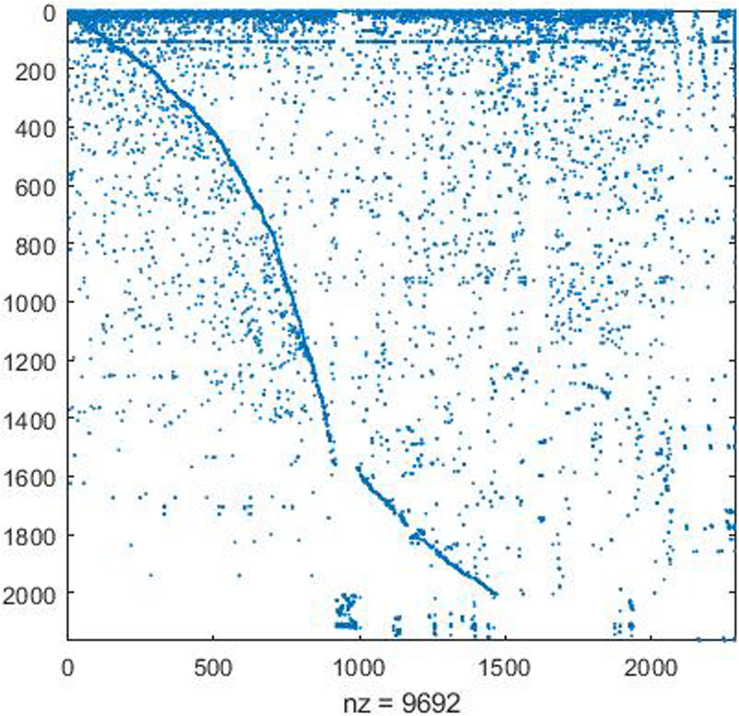
Visualization of stoichiometric matrix.

The reconstructed network was collated according to the format required by the COBRA toolbox and imported into the MATLAB platform using the COBRA toolbox. The stoichiometric matrix formed by model transformation is shown in Figure 1. To test the model’s ability to synthesize biomass precursors, the exchange reactions of the precursor substances such as amino acids, fatty acids, carbohydrates, and nucleic acids were set as the objective function and simulated by the FBA algorithm, respectively. After simulations, the maximum fluxes of glutamate, cytidylic acid (CMP), and 15 fatty acids were found to be 0. It indicates that there are some flaws in the synthetic route of these metabolites in the model, which need to be revised.

Referencing to the glutamate synthesis pathway in the KEGG database, the metabolic flow of glutamate synthesis in the model was studied. It was found that reactions R02073 and R01518 necessary for the synthesis of glutamate were missing in the model, and the reactants and products of reaction R00200 were in the wrong direction. Therefore, R02073 and R01518 were added to the network, and the reactants and products of R00200 were exchanged. In the recalculation, the flux of the exchange reaction of glutamate was not 0; that is, glutamate could be synthesized by the model.

The synthesis route of CMP and fatty acids was analyzed in the same way. After adding reactions R00158, R00512, and R00515 in the pyrimidine metabolism system, CMP could be synthesized. In the metabolic flow analysis of fatty acids, it was found that the metabolic pathway of fatty acids in the model was extremely incomplete. Finally, 29 reactions in the biosynthesis route of unsaturated fatty acids were added to make all the fatty acids that can be synthesized.

After the synthesis of all the precursors verified, the capability of five commercial varieties to synthesize biomass was checked, respectively. It was found that the biomass of all the five varieties was able to be synthesized. The characteristics of the final GSMN of *L. vannamei*, namely, *i*GH3005 (Supplementary File 3), are shown in Table 3.

**TABLE 3:** The characteristics of *i*GH3005.

**Item**	**Count**
Genes	3,005
Reaction	2,292
Metabolic reaction	2,110
Transport reaction	91
Exchange reaction	91
Biomass reaction	1
Metabolite	2,184
Cytosol metabolite	2,091
Extracellular metabolite	93
Pathway	132
Subsystem	12

The maximum biomass synthesis rates (i.e., growth rate) for the five *L. vannamei* commercial varieties were simulated with *i*GH3005, respectively (Table 4). Riyekuai has the highest growth rate, Guangtai was in the middle, and Lutai, Kehai, and Puruiying grow slowest, which is consistent with the result of average weights obtained during sampling.

**TABLE 4:** The maximum growth rate of the five *L. vannamei* commercial varieties.

**Commercial varieties**	**Maximum growth rate**
Lutai	2.32
Riyekuai	3.41
Kehai	2.83
Guangtai	3.04
Puruiying	2.35

### Analysis on Nutritional Requirements of Five *Litopenaeus vannamei* Commercial Varieties

#### Demand Analysis of Protein

For aquatic animals, protein in feed is almost the only nitrogen source used for cellular protein synthesis. Protein is not only the primary material to produce new cells and make up for old cells but also an essential component of various tissues, enzymes, and hormones. Lack of protein in the feed will cause a series of physiological and biochemical reaction obstacles, which will affect the growth and survival of shrimp. However, the high protein levels in feed will not only affect the development of shrimp, pollute the environment, but also increase the cost of feed and cause waste (Cuzon et al., 2004).

The protein intake from feed can only be absorbed and utilized by the body after being digested, decomposed into peptides, amino acids, and other small molecular compounds in the digestive tract (Marangos et al., 1989). Therefore, the protein demand for shrimp is essentially the demand for amino acids (Cuzon et al., 2004). Amino acids can be divided into non-essential amino acids and essential amino acids. Essential amino acids refer to the amino acids that cannot be synthesized by *L. vannamei* itself or the amount of synthesis is quite small, which cannot meet the needs of growth of *L. vannamei* and must be provided by feed. The appropriate content and proportion of essential amino acids in feed are conducive to make proteins and feeds work at full capacity, which is helpful for the growth of shrimps (Wang, 1991).

*Litopenaeus vannamei* needs 10 essential amino acids, including arginine, lysine, methionine, leucine, isoleucine, histidine, phenylalanine, threonine, tryptophan, and valine (Kai et al., 2003). Kai et al. (2003) reported the demand for essential amino acids in shrimp feed. We transformed the unit into mmolgDW^–1^h^–1^ and compared it with the simulation results of *i*GH3005 (Table 5). The simulation results are generally higher than the requirements provided in literature, which indicates that the requirement of these essential amino acids for *L. vannamei* might be greater than that mentioned in the literature. Therefore, there is still room to increase the protein content containing these essential amino acids in feed. Actually, feed producers usually decrease the proportion of proteins in feed to reduce the feed cost and avoid the environmental pollution, which makes the protein content in feed lower than the theoretical value. In addition, different growth stages of shrimp in different culture environments have different requirements for essential amino acids, which may lead to the deviation of the results.

**TABLE 5:** Requirements of essential amino acids by *L. vannamei* in literature and simulation results.

**Amino acids**	**Requirement %**	**Requirement mmolgDW^–1^h^–1^**	**Simulation results**				
			**Lutai**	**Riyekuai**	**Kehai**	**Guangtai**	**Puruiying**
Arginine	2.43 (Kai et al., 2003)	0.14	−5.00	−2.94	−5.00	−3.23	−3.24
Lysine	0.63 (Kai et al., 2003)	0.04	−0.11	−0.10	−0.11	−0.11	−0.10
Methionine	1.73 (Kai et al., 2003)	0.13	−0.26	−0.24	−0.26	−0.25	−0.25
Leucine	2.18 (Kai et al., 2003)	0.17	−0.55	−0.45	−0.54	−0.47	−0.48
Isoleucine	2.51 (Kai et al., 2003)	0.17	−0.45	−0.43	−0.44	−0.44	−0.43
Histidine	0.73 (Kai et al., 2003)	0.05	−0.16	−0.15	−0.15	−0.15	−0.15
Phenylalanine	1.28 (Kai et al., 2003)	0.08	−5.00	−5.00	−5.00	−5.00	−5.00
Threonine	1.15 (Kai et al., 2003)	0.10	−5.00	−5.00	−5.00	−5.00	−5.00
Tryptophan	0.28 (Yongli, 2013)	0.01	−0.03	−0.03	−0.03	−0.02	−0.03
Valine	1.35 (Kai et al., 2003)	0.12	−0.31	−0.30	−0.30	−0.30	−0.29

From Table 5, we can also see that Lutai and Kehai require more arginine than the other three commercial varieties. According to the simulation results, the requirement of arginine in Lutai and Kehai is 5 mmolgDW^–1^h^–1^ and that in Riyekuai, Guangtai, and Puruiyin are 2.94, 3.23, and 3.24 mmolgDW^–1^h^–1^, respectively. The arginine requirements in Lutai and Kehai are the same, while it is about 1.54∼1.7 times compared with the other three commercial varieties. Therefore, the arginine content in the diet of Lutai and Kehai should be 54%∼70% higher than the other three commercial varieties. However, in the actual breeding process, the feeds for all the commercial varieties are the same, which may be the reason for the low average body weight of Lutai and Kehai under the same breeding conditions.

#### Demand Analysis of Lipid

Lipid is an essential energy substance in growth and development, providing essential fatty acids and other nutrients. The insufficient content of lipid in feed can lead to a decrease of protein utilization rate, disorder of shrimp metabolism, as well as lack of fat-soluble vitamins and essential fatty acids.

The essential fatty acids of *L. vannamei* are linoleic acid (LOA), linolenic acid (LNA), eicosapentaenoic acid (EPA), and docosahexaenoic acid (DHA) (Gaowang et al., 2018). Previous studies have shown that LOA can be transformed to γ-LNA catalyzed by △6-dehydrogenase, and then γ-LNA turned into arachidonic acid (Xinzhen et al., 2008). EPA and DHA can be metabolized by α-LNA (Cook and Mcmaster, 2002). Gonzalez-Felix et al. (2002) found that the growth performance of *L. vannamei* was better promoted by the addition of long-chain high unsaturated fatty acids in the feed. Among them, the minimum amounts of EPA and DHA were shown in Table 6. They also evaluated the nutritional value of short-chain polyunsaturated fatty acids LOA and LNA in *L. vannamei*. The results showed that there was no significant difference between the treatment groups with different dosages (Gonzalez-Felix et al., 2002). We compared the simulation results of the five *L. vannamei* commercial varieties on the demand of essential fatty acids with the work of Gonzalez-Felix et al. (2002) (Table 6).

**TABLE 6:** Requirements of fatty acids by *L. vannamei* in literature and simulation results.

**Fatty Acids**	**Requirement**	**Requirement mmolgDW^–1^h^–1^**	**Simulation results**				
	**g/100 g**		**Lutai**	**Riyekuai**	**Kehai**	**Guangtai**	**Puruiying**
γ-LNA	N/A	N/A	0.00	4.88	0.00	0.00	0.00
LNA	N/A	N/A	−0.01	−0.01	−0.01	−0.01	−0.01
EPA	0.20 (Gonzalez-Felix et al., 2002)	0.01	−0.02	−0.01	−0.01	−0.01	−0.01
DHA	0.30 (Gonzalez-Felix et al., 2002)	0.01	4.86	−0.02	4.85	−0.02	−0.02
LOA	N/A	N/A	−0.04	−0.04	−0.04	−0.05	−0.05

*DHA, docosahexaenoic acid; EPA, eicosapentaenoic acid; LNA, linolenic acid; LOA, linoleic acid.*

The theoretical demands for EPA and DHA of the five *L. vannamei* commercial varieties are higher than that in literature. It indicates that the addition of EPA and DHA in feed is conducive to the growth of shrimp, which is consistent with the research results of Gonzalez-Felix et al. (2002), but the amount may need to be increased. It can also be seen from Table 6 that the accumulation of γ-LNA by Riyekuai and DHA accumulation by Lutai and Kehai are abnormally large. Through the metabolic flow analysis on the accumulation process of γ-LNA in *L. vannamei*, we found that the flux of reaction R08181 (gamma-Linolenoyl-CoA + H_2_O <=> CoA + (6Z, 9Z, 12Z)-Octadecatrienoic acid) reached 4.88 mmol/gDW^–1^h^–1^. The metabolite flow analysis of DHA accumulation revealed that the fluxes of the reaction related to DHA synthesis R08180: [(4Z, 7z, 10z, 13z, 16Z, 19Z)-docosahexaenoyl COA + H_2_O <=> COA + (4Z, 7z, 10z, 13z, 16Z, 19Z)-docosahexaenoic acid] were 4.88 and 4.876 mmolgDW^–1^h^–1^ in Lutai and Kehai, respectively.

The corresponding genes of R08180 and R08181 were both pvm:113803399 and pvm:113804504. By searching the KEGG database, we found that their corresponding enzyme was tyrosine-protein kinase src64b like, which means that this enzyme in different commercial varieties of *L. vannamei* has not only different expression levels but also catalyzes different reactions. The enzyme catalyzes reaction R08181 to rapidly synthesize γ-LNA in Riyekuai, while in Lutai and Kehai, the enzyme catalyzes R08180 to accumulate large amounts of DHA. According to the sampling results in Table 4, the average weight of Riyekuai is the largest, while Kehai and Lutai are lower. Therefore, we speculated that the catalytic direction of this enzyme may significantly impact the growth of shrimp.

#### Demand Analysis of Mineral Elements

Minerals, including macro-elements and trace elements, are indispensable nutrients for the growth, development, and reproduction of shrimp. The research on the nutritional, physiological function and demand of shrimp minerals plays a positive role in promoting the growth and development of shrimp. Although shrimps can absorb some minerals from water, they generally lose many minerals due to molting. Therefore, a certain amount of minerals must be provided by feed. Researchers have achieved some results in the utilization of trace elements in *L. vannamei*. We compared the simulation results of *i*GH3005 with this literature (Table 7).

**TABLE 7:** Requirements of mineral element by *L. vannamei* in literature and simulation results.

**Mineral element**	**Requirement %**	**Requirement mmolgDW^–1^h^–1^**	**Simulation results**				
			**Lutai**	**Riyekuai**	**Kehai**	**Guangtai**	**Puruiying**
Ca	0.80% (Kai et al., 2004)	0.00	−0.03	−0.03	−0.04	−0.04	−0.07
P	1.20% (Kai et al., 2004)	0.00	−0.38	−0.36	−0.37	−0.38	−0.39
K	0.79% (Subi et al., 2016)	0.00	−0.15	−0.20	−0.20	−0.20	−0.17
Mg	0.12 g/100 g (Davis et al., 2010)	0.05	−0.06	−0.06	−0.06	−0.06	−0.07
Zn	0.0059 g/100 g (Haitao et al., 2017)	0.00	−0.00	−0.00	−0.00	−0.00	−0.00
Cu	0.0034 g/100 g (Davis et al., 2010)	0.00	−0.00	−0.00	−0.00	−0.00	−0.00
Fe	N/A	N/A	−0.00	−0.00	−0.00	−0.00	−0.00
Mn	0.015 g/100 g (Xian et al., 2013)	0.00	−0.00	0.00	−0.00	0.00	−0.00

The requirements of zinc, magnesium, and copper in the simulation results are very close to literature (Davis et al., 2010; Haitao et al., 2017), reflecting the high quality of *i*GH3005 in the mineral simulations. The calculated theoretical values of calcium and phosphorus for *L. vannamei* are different from literature. It may be because calcium and phosphorus are two of the main water pollutants, and the amount of calcium and phosphorus in feed has a specific limit (Briggs and Fvnge-Smith, 1994).

In addition, in the simulation results of *i*GH3005, the demand of potassium and iron for *L. vannamei* is significantly higher than that reported in literature, and the demand of manganese was lower than that of actual feed. Therefore, we suggest that the amount of potassium and iron in the feed can be increased appropriately, and the amount of manganese can be reduced to improve the yield of *L. vannamei*.

#### Demand Analysis of Carbohydrate

The disaccharides or polysaccharides ingested by crustaceans need to be hydrolyzed into glucose and then transported through the hemolymph and absorbed by different tissues for glucose and glycogen metabolism (Wang et al., 2015). The unneeded carbohydrate in the body is converted into glycogen or fat for storage mainly in hepatopancreas and muscle of *L. vannamei*, providing energy for growth, molting, and reproduction. There are some corresponding enzymes in the digestive tract of shrimp, such as α-amylase, α-glucosidase, α-maltase, α-sucrase, galactosidase, chitinase, chitosanase, and cellulase, so shrimp can digest different kinds of carbohydrates (Glass and Stark, 1995). However, the carbohydrate digestibility of aquatic animals depends on the structure and complexity of carbohydrate (Bergot, 1979; Spannhof and Plantikow, 1983). Relatively complex carbohydrates can only be digested after enzymatic hydrolysis, so they are not as easy to digest as monosaccharides (Alava and Pascual, 1987; Shiau and Peng, 1992).

*i*GH3005 was used to simulate the requirement of lactose, glucose, maltose, sucrose, and fructose (Table 8). The simulated lactose accumulation of Lutai is extremely large. Through the metabolic flow analysis of the lactose accumulation process, it is found that the flux of reaction R00503 (UDP-alpha-D-galactose + D-glucose < = > UDP + lactose) reaches 4.878 mmolgDW^–1^h^–1^ and that of other commercial varieties is 0. The corresponding genes of R00503 were pvm:113828354 (LOC113828354), pvm:113828357 (LOC113828357), pvm:113828366 (LOC113828366), pvm:113828370 (LOC113828370), pvm:113828374 (LOC113828374), and pvm:113828381 (loc113828381). We speculated that the high expression level of these genes in Lutai resulted in the accumulation of lactose. In addition, in the simulation results, the glucose demand of Lutai is greater than that of the other four commercial varieties. By studying the metabolic process of glucose, we found that the flux of alpha-D-glucose producing reaction R01602 (beta-D-glucose alpha-D-glucose) in Lutai network was lower than that of the other four varieties. In comparison, the flux of alpha-D-glucose consuming reaction R01788 (alpha-D-glucose + orthophosphate alpha-D-glucose 6-phosphate + H_2_O) was higher than that of the other four varieties, which resulted in the much more glucose demand in Lutai than the other four varieties. Based on the simulation, the gene PVM:113819971 corresponding to R01602 has a low expression level, and the gene PVM:113819863 corresponding to R01788 has a high expression level in Lutai, which points out the direction for the further wet experiment.

**TABLE 8:** The simulation results of the requirements for carbohydrates in *L. vannamei.*

**Carbohydrate**	**Simulation results**				
	**Lutai**	**Riyekuai**	**Kehai**	**Guangtai**	**Puruiying**
Lactose	4.87	−0.01	−0.01	−0.01	−0.01
Glucose	−3.50	2.45	2.33	7.23	7.18
Maltose	−0.01	−0.01	−0.01	−0.01	−0.01
Sucrose	−0.03	−0.03	−0.04	−0.04	−0.04
Fructose	−5.00	−5.00	−5.00	−5.00	−5.00

In addition, in the simulation results, the five commercial varieties of *L. vannamei* all have a small demand for sucrose and maltose. Therefore, we suggest that sucrose and maltose can be added appropriately in feed. We can also see that the demand for glucose of Lutai is larger than that of the other four varieties, and the demand for lactose in Riyekuai, Kehai, Guangtai, and Puiying is larger than that in Lutai. Hence, we advise that carbohydrate content in the feed for different commercial varieties of *L. vannamei* should be adjusted. The appropriate amount of glucose can be supplied to Lutai, and some lactose can be provided to the other four varieties. It should be noted that at the experimental level, glucose can be directly added to shrimp feed as a carbohydrate source (Ran, 2007), while at the industrial level, from the perspective of cost reduction, other alternatives are better, such as corn starch, wheat starch, potato starch, and dextrin. The amount of alternatives can be added according to the simulation results and the conversion efficiency of alternatives to glucose. In addition, in the simulation results, the demand of fructose for the five commercial varieties of *L. vannamei* is abnormally large, which may be due to the lack of key speed limiting reaction in the model.

## Conclusion

Genome-scale metabolic network has become an indispensable tool for systematic research on metabolism and applied to many microorganisms and model organisms. Based on the genomic data of *L. vannamei* and KEGG database, the first GSMN of *L. vannamei i*GH3005 was reconstructed. The biomass equations of five *L. vannamei* commercial varieties were further constructed based on the nutritional composition detection and added to the model. The optimal nutrient requirements of the five *L. vannamei* commercial varieties were simulated. Based on the simulation results, we discussed the different expressions of some important genes and put forward some suggestions on the improvement of *L. vannamei* feed. We found that tyrosine-protein kinase src64b like has not only different expression levels but also catalyzed different reactions in different commercial varieties of *L. vannamei*. In addition, each shrimp race has a different carbohydrate usage probably due to differences in gene expression of glycolytic genes. We suggest that EPA, DHA, potassium, iron, sucrose, and maltose should be added to the feed of all five varieties. Reducing the amount of manganese could also promote the growth of *L. vannamei*. This investigation suggests that a rational and targeted modification in the macronutrient content of shrimp feed would lead to an increase in growth and feed conversion rate.

## Data Availability Statement

The original contributions presented in the study are included in the article/Supplementary Material, further inquiries can be directed to the corresponding author/s.

## Ethics Statement

The study was approved by the College of Life Sciences, Tianjin Normal University.

## Author Contributions

CG analyzed the data and wrote the manuscript. JY and JL organized the data. JS and TH designed the experiment and revised the manuscript. All authors contributed to the article and approved the submitted version.

## Conflict of Interest

The authors declare that the research was conducted in the absence of any commercial or financial relationships that could be construed as a potential conflict of interest.

## Supplementary Material

Supplementary File 1 The results of Nutritional composition detection for the five commercial varieties of *L. vannamei*.

Supplementary File 2 The biomass reactions for the five commercial varieties of *L. vannamei*.

Supplementary File 3 The reconstructed model *i*GH3005.

Supplementary File 4 The detail calculation processes of network evaluation and nutrient requirement analysis.
