# Supplementary material for: Reconstruction of Litopenaeus vannamei Genome-Scale Metabolic Network Model and Nutritional Requirements Analysis of Different Shrimp Commercial Varieties
Source: Front Genet. 2021 May 12;12:658109. doi: 10.3389/fgene.2021.658109 (PMC8149995; doi:10.3389/fgene.2021.658109)
Supplement: Supplementary File 3 — The reconstructed model iGH3005. [file Table_3.DOCX]

**1.** **The biomass reactions for Lutai：**

163.8923 C00001[c] + 0.0900 C00267[c] + 0.0219 C00095[c] + 0.0115 C00089[c] + 0.0115 C00243[c] + 0.0115 C00208[c] + 0.3777 C19162[c] + 0.0300 C00076[c] + 0.0004 C00070[c] + 0.0007 C00023[c] + 0.4307 C00238[c] + 0.0610 C00305[c] + 0.0001 C00034[c] + 0.1544 C01330[c] + 0.0009 C00038[c] + 0.0002 C00055[c] + 0.0002 C00020[c] + 0.0006 C00105[c] + 0.0004 C00144[c] + 0.0226 C00130[c] + 0.6121 C00049[c] + 0.2601 C00188[c] + 0.2956 C00065[c] + 0.9072 C00025[c] + 0.7798 C00037[c] + 0.5582 C00041[c] + 0.3091 C00183[c] + 0.1556 C00073[c] + 0.2585 C00407[c] + 0.5502 C00123[c] + 0.1545 C00082[c] + 0.2112 C00079[c] + 0.4493 C00047[c] + 0.7370 C00014[c] + 0.1075 C00135[c] + 0.4727 C00062[c] + 0.3722 C00148[c] + 0.0348 C00078[c] + 0.0871 C00097[c] + 0.0003 C02679[c] + 0.0002 M00001[c] + 0.0038 C06424[c] + 0.0023 C16537[c] + 0.0535 C00249[c] + 0.0127 C08362[c] + 0.0193 C01530[c] + 0.0299 C00712[c] + 0.0413 C01595[c] + 0.0013 C06425[c] + 0.0062 C06427[c] + 0.0015 C08281[c] + 0.0006 C03242[c] + 0.0004 C08316[c] + 0.0061 C00219[c] + 0.0004 C08323[c] + 0.0209 C06429[c] + 0.0029 M00002[c] + 0.0008 M00003[c] + 0.0024 C16526[c] + 0.0003 M00004[c] + 0.0027 C16525[c] + 0.0016 C16522[c] + 0.0006 M00005[c] + 0.0155 C06428[c] + 29.8303 C00002[c] --> 29.8303 C00008[c] + 29.8303 C00009[c] + 29.8303 C00080[c]

**2.** **The biomass reactions for Riyekuai：**

157.1674 C00001[c] + 0.1112 C00267[c] + 0.0213 C00095[c] + 0.0112 C00089[c] + 0.0112 C00243[c] + 0.0112 C00208[c] + 0.3600 C19162[c] + 0.0300 C00076[c] + 0.0005 C00070[c] + 0.0006 C00023[c] + 0.2930 C00238[c] + 0.0569 C00305[c] + 0.1983 C01330[c] + 0.0009 C00038[c] + 0.0001 C00055[c] + 0.0001 C00020[c] + 0.0005 C00105[c] + 0.0003 C00144[c] + 0.0205 C00130[c] + 0.5944 C00049[c] + 0.2461 C00188[c] + 0.2756 C00065[c] + 0.8709 C00025[c] + 0.7404 C00037[c] + 0.5270 C00041[c] + 0.2964 C00183[c] + 0.1508 C00073[c] + 0.2440 C00407[c] + 0.4462 C00123[c] + 0.1508 C00082[c] + 0.2030 C00079[c] + 0.4273 C00047[c] + 0.6801 C00014[c] + 0.1036 C00135[c] + 0.4476 C00062[c] + 0.3884 C00148[c] + 0.0319 C00078[c] + 0.0806 C00097[c] + 0.0003 C02679[c] + 0.0037 C06424[c] + 0.0022 C16537[c] + 0.0518 C00249[c] + 0.0123 C08362[c] + 0.0187 C01530[c] + 0.0290 C00712[c] + 0.0401 C01595[c] + 0.0012 C06425[c] + 0.0060 C06427[c] + 0.0014 C08281[c] + 0.0006 C03242[c] + 0.0004 C08316[c] + 0.0060 C00219[c] + 0.0004 C08323[c] + 0.0202 C06429[c] + 0.0032 M00002[c] + 0.0008 M00003[c] + 0.0040 C16526[c] + 0.0004 M00004[c] + 0.0045 C16525[c] + 0.0020 C16522[c] + 0.0009 M00005[c] + 0.0128 C06428[c] + 29.8303 C00002[c] --> 29.8303 C00008[c] + 29.8303 C00009[c] + 29.8303 C00080[c]

**3.** **The biomass reactions for Kehai：**

176.7442 C00001[c] + 0.0497 C00267[c] + 0.0232 C00095[c] + 0.0122 C00089[c] + 0.0122 C00243[c] + 0.0122 C00208[c] + 0.3661 C19162[c] + 0.0419 C00076[c] + 0.0006 C00070[c] + 0.0019 C00023[c] + 0.3531 C00238[c] + 0.0608 C00305[c] + 0.0001 C00034[c] + 0.1984 C01330[c] + 0.0009 C00038[c] + 0.0002 C00055[c] + 0.0006 C00020[c] + 0.0005 C00105[c] + 0.0002 C00144[c] + 0.0148 C00130[c] + 0.6182 C00049[c] + 0.2589 C00188[c] + 0.2965 C00065[c] + 0.9066 C00025[c] + 0.8515 C00037[c] + 0.5849 C00041[c] + 0.3047 C00183[c] + 0.1539 C00073[c] + 0.2567 C00407[c] + 0.5376 C00123[c] + 0.1554 C00082[c] + 0.2132 C00079[c] + 0.4442 C00047[c] + 0.7033 C00014[c] + 0.1067 C00135[c] + 0.4547 C00062[c] + 0.4109 C00148[c] + 0.0348 C00078[c] + 0.0860 C00097[c] + 0.0003 C02679[c] + 0.0040 C06424[c] + 0.0024 C16537[c] + 0.0566 C00249[c] + 0.0134 C08362[c] + 0.0205 C01530[c] + 0.0317 C00712[c] + 0.0437 C01595[c] + 0.0014 C06425[c] + 0.0066 C06427[c] + 0.0015 C08281[c] + 0.0006 C03242[c] + 0.0004 C08316[c] + 0.0065 C00219[c] + 0.0005 C08323[c] + 0.0221 C06429[c] + 0.0008 C08320[c] + 0.0025 M00002[c] + 0.0005 M00003[c] + 0.0020 C16526[c] + 0.0003 M00004[c] + 0.0020 C16525[c] + 0.0007 C16522[c] + 0.0006 M00005[c] + 0.0110 C06428[c] + 29.8303 C00002[c] --> 29.8303 C00008[c] + 29.8303 C00009[c] + 29.8303 C00080[c]

**4. The biomass reactions for Guangtai：**

183.7519 C00001[c] + 0.0639 C00267[c] + 0.0239 C00095[c] + 0.0126 C00089[c] + 0.0126 C00243[c] + 0.0126 C00208[c] + 0.3785 C19162[c] + 0.0445 C00076[c] + 0.0006 C00070[c] + 0.0006 C00023[c] + 0.3285 C00238[c] + 0.0621 C00305[c] + 0.2006 C01330[c] + 0.0009 C00038[c] + 0.0001 C00055[c] + 0.0003 C00020[c] + 0.0004 C00105[c] + 0.0002 C00144[c] + 0.0146 C00130[c] + 0.6007 C00049[c] + 0.2501 C00188[c] + 0.2862 C00065[c] + 0.8885 C00025[c] + 0.8555 C00037[c] + 0.5669 C00041[c] + 0.2990 C00183[c] + 0.1527 C00073[c] + 0.2517 C00407[c] + 0.4744 C00123[c] + 0.1543 C00082[c] + 0.2096 C00079[c] + 0.4373 C00047[c] + 0.7141 C00014[c] + 0.1039 C00135[c] + 0.4500 C00062[c] + 0.3192 C00148[c] + 0.0238 C00078[c] + 0.0781 C00097[c] + 0.0041 C06424[c] + 0.0025 C16537[c] + 0.0583 C00249[c] + 0.0138 C08362[c] + 0.0211 C01530[c] + 0.0327 C00712[c] + 0.0451 C01595[c] + 0.0014 C06425[c] + 0.0068 C06427[c] + 0.0016 C08281[c] + 0.0006 C03242[c] + 0.0067 C00219[c] + 0.0005 C08323[c] + 0.0228 C06429[c] + 0.0008 C08320[c] + 0.0024 M0002[c] + 0.0004 M0003[c] + 0.0014 C06425[c] + 0.0003 M07776[c] + 0.0019 C06584[c] + 0.0012 C06384[c] + 0.0006 M07226[c] + 0.0094 C06854[c] + 29.8303 C00002[c] --> 29.8303 C00008[c] + 29.8303 C00009[c] + 29.8303 C00080[c]

**5. The biomass reactions for Puruiying：**

184.7876 C00001[c] + 0.1050 C00267[c] + 0.0240 C00095[c] + 0.0126 C00089[c] + 0.0126 C00243[c] + 0.0126 C00208[c] + 0.3871 C19162[c] + 0.0696 C00076[c] + 0.0005 C00070[c] + 0.0008 C00023[c] + 0.4263 C00238[c] + 0.0654 C00305[c] + 0.0001 C00034[c] + 0.1712 C01330[c] + 0.0009 C00038[c] + 0.0003 C00055[c] + 0.0003 C00020[c] + 0.0007 C00105[c] + 0.0003 C00144[c] + 0.0065 C00130[c] + 0.5891 C00049[c] + 0.2448 C00188[c] + 0.2763 C00065[c] + 0.8641 C00025[c] + 0.8080 C00037[c] + 0.5651 C00041[c] + 0.2937 C00183[c] + 0.1482 C00073[c] + 0.2461 C00407[c] + 0.4835 C00123[c] + 0.1532 C00082[c] + 0.2025 C00079[c] + 0.4264 C00047[c] + 0.7125 C00014[c] + 0.1010 C00135[c] + 0.4428 C00062[c] + 0.3375 C00148[c] + 0.0283 C00078[c] + 0.0766 C00097[c] + 0.0041 C06424[c] + 0.0025 C16537[c] + 0.0586 C00249[c] + 0.0139 C08362[c] + 0.0212 C01530[c] + 0.0328 C00712[c] + 0.0453 C01595[c] + 0.0014 C06425[c] + 0.0068 C06427[c] + 0.0016 C08281[c] + 0.0006 C03242[c] + 0.0067 C00219[c] + 0.0005 C08323[c] + 0.0229 C06429[c] + 0.0008 C08320[c] + 0.0004 C08316[c] + 0.0024 M00002[c] + 0.0005 M00003[c] + 0.0018 C16526[c] + 0.0002 M00004[c] + 0.0016 C16525[c] + 0.0013 C16522[c] + 0.0006 M00005[c] + 0.0011 C16533[c] + 0.0095 C06428[c] + 29.8303 C00002[c] --> 29.8303 C00008[c] + 29.8303 C00009[c] + 29.8303 C00080[c]
