# Supplementary material for: Reconstruction of Litopenaeus vannamei Genome-Scale Metabolic Network Model and Nutritional Requirements Analysis of Different Shrimp Commercial Varieties
Source: Front Genet. 2021 May 12;12:658109. doi: 10.3389/fgene.2021.658109 (PMC8149995; doi:10.3389/fgene.2021.658109)
Supplement: Supplementary File 4 — The detail calculation processes of network evaluation and nutrient requirement analysis. [file Table_4.DOCX]

1. ***Network evaluation***

The network evaluation includes the following four steps: (1) Initialization of the COBRA toolbox; (2) Importation of a model; (3) Visualization of a model; (4) Calculation of the precursor synthesis; (5) Calculation of biomass. All operation commands are executed in MATLAB, the specific operation command is as follows:

**(1) Initialization of the** **COBRA toolbox:**

*>>initCobraToolbox*

Every time we open Matlab, we need to execute the command to initialize COBRA toolbox, load corresponding functions, update the changed or newly added functions in GitHub public library, and ensure that the latest version of Cobra toolbox is used every time we process the model. After initialization, "The COBRA Toolbox is up-to-date" is displayed.

**(2) Importation of a model**

>>model=readCbModel(filename)

Parameter “*filename*” is the storage path and the name of the file; When the “*filename*” parameter is left blank, the folder selection window will appear automatically and the file imported should be selected manually. In this work, we input the command: *> > model = readcbmodel ()*, the folder selection window appeared, and the model file was selected manually.

1. **Visualization of a model**

*>>spy(model.S)*

The model imported is a stoichiometric matrix of metabolites and reactions, which can be represented graphically by this command. In this work, we entered this command and got Figure 1.

1. **Calculation of the precursor synthesis**

*>>FBAsolution=optimizeCbModel(model,osenseStr)*

*“optimizeCbModel”* function was used to evaluate the model with FBA algorithm. Parameter “*osenseStr”* is used to maximize or minimize the objective function, and its default value is 'Max'. In this work, to evaluate the model, we calculated the synthesis of all the precursors in biomass equation. Firstly, we wrote all the precursors in a new excel file and put the file in the COBRA installation folder; then input the command:*>>[num,txt]=xlsread('C:/gcc/cobratoolbox/biomass.xlsx')* to read the file into Matlab; finally, the synthesis flux of each biomass was calculated. The programming is as follows:

*i=1*

*F=[]*

*for i=1:63 ‘ 63 is the number of precursors*

*d=[txt{i},'[c]']*

*model=addDemandReaction(model,d)*

*e=['DM_',d]*

*model=changeObjective(model,e)*

*FBAsolution=optimizeCbModel(model,'max')*

*F=[F,FBAsolution.f]*

*end*

*format long g*

*B=reshape(F,63,1)*

1. **Calculation of biomass**

The biomass equation was set to be the objective function, and the maximum value of biomass synthesis is calculated with FBA algorithm. The results are shown in Table 4. The programming is as follows:

*>>model=changeObjective(model,'B00001')*

*>>FBAsolution = optimizeCbModel(model)*

1. ***Nutrient requirement analysis***

The nutrient requirement analysis includes four steps: (1) Initialization of the COBRA toolbox; (2) Importation of a model; (3) Computing a flux balance analysis solution; (4) Exportation of results. The programming is as follows:

1. **Initialization of the Cobra toolbox:**

*>>initCobraToolbox*

1. **Importation of a model**

*>>model=readCbModel()*

1. **Computing a flux balance analysis solution**

The biomass equation was set to be the objective function and its flux was fixed to be 1 gDW^-1^h^-1^ in the model file. Then FBA algorithm was used to calculate the flux of nutrients requirement.

*>>model=changeObjective(model,'B00001')*

*>>FBAsolution = optimizeCbModel(model)*

**4.** **Exportation of results**

*>>printFluxVector(model, fluxData, nonZeroFlag, excFlag)*

Parameter “*fluxData*” is the data matrix; “*nonZeroFlag*” means only output non-zero flux, the default value is false; “*excFlag*” means only output exchange reaction flux, the default value is false; In this work, we input the command: *>>printFluxVector(model, FBAsolution.v, false, true)*, then the flux of all exchange reactions was output, that is, all the nutrients requirement of *L.vannamei*.
